# Temperature alters the inotropic, chronotropic and proarrhythmic effects of histamine in atrial muscle preparations from humans and H_2_-receptor overexpressing mice

**DOI:** 10.1007/s00210-023-02457-x

**Published:** 2023-03-23

**Authors:** Robert J. R. Hoffmann, Ulrich Gergs, Britt Hofmann, Uwe Kirchhefer, Joachim Neumann

**Affiliations:** 1 Institut Für Pharmakologie und Toxikologie, Medizinische Fakultät, Martin-Luther-Universität Halle-Wittenberg, 06097 Halle, Germany; 2 Medizinische Fakultät, Herzchirurgie, Martin-Luther-Universität Halle-Wittenberg, 06097 Halle, Germany; 3 Institut für Pharmakologie und Toxikologie, Medizinische Fakultät, Westfälische Wilhelms-Universität, Domagkstr. 12, 48149 Münster, Germany

**Keywords:** Histamine, H_2_ receptor, Hypothermia, Hyperthermia, Arrhythmia, Inotropy, Chronotropy, Human phospholamban

## Abstract

We investigated whether hypothermia and hyperthermia can alter the efficacy and potency of histamine at increasing the force of cardiac contractions in mice that overexpress the human H_2_ receptor only in their cardiac myocytes (labelled H_2_-TG). Contractile studies were performed in an organ bath on isolated, electrically driven (1 Hz) left atrial preparations and spontaneously beating right atrial preparations from H_2_-TG mice and wild-type (WT) littermate control mice. The basal beating rate in the right atrial preparations from H_2_-TG mice was lowered by hypothermia (23 °C) and elevated by hyperthermia (42 °C). Furthermore, the efficacy of histamine (0.01–100 µM) at exerting positive inotropic effects was more severely attenuated in the left and right H_2_-TG mouse atria under hypothermia and hyperthermia than under normothermia (37 °C). Similarly, the inotropic response to histamine was attenuated under hypothermia and hyperthermia in isolated electrically stimulated (1 Hz) right atrial preparations obtained from humans undergoing cardiac surgery. The phosphorylation state of phospholamban at serine 16 at 23 °C was inferior to that at 37 °C in left atrial preparations from H_2_-TG mice in the presence of 10 µM histamine. In contrast, in human atrial preparations, the phosphorylation state of phospholamban at serine 16 in the presence of 100 µM histamine was lower at 42 °C than at 37 °C. Finally, under hyperthermia, we recorded more and longer lasting arrhythmias in right atrial preparations from H_2_-TG mice than in those from WT mice. We conclude that the inotropic effects of histamine in H_2_-TG mice and in human atrial preparations, as well as the chronotropic effects of histamine in H_2_-TG mice, are temperature dependent. Furthermore, we observed that, even without stimulation of the H_2_ receptors by exogenous agonists, temperature elevation can increase arrhythmias in isolated right atrial preparations from H_2_-TG mice. We propose that H_2_ receptors play a role in hyperthermia-induced supraventricular arrhythmias in human patients.

## Introduction

In the human heart, histamine simultaneously exerts a positive inotropic effect (PIE) and a positive chronotropic effect (PCE) on the organ, augmenting the rate of tension development and the rate of tension relaxation and decreasing the time to tension relaxation (e.g. Neumann et al. 2021a, review: Neumann et al. 2021b).

**Fig. 1:**
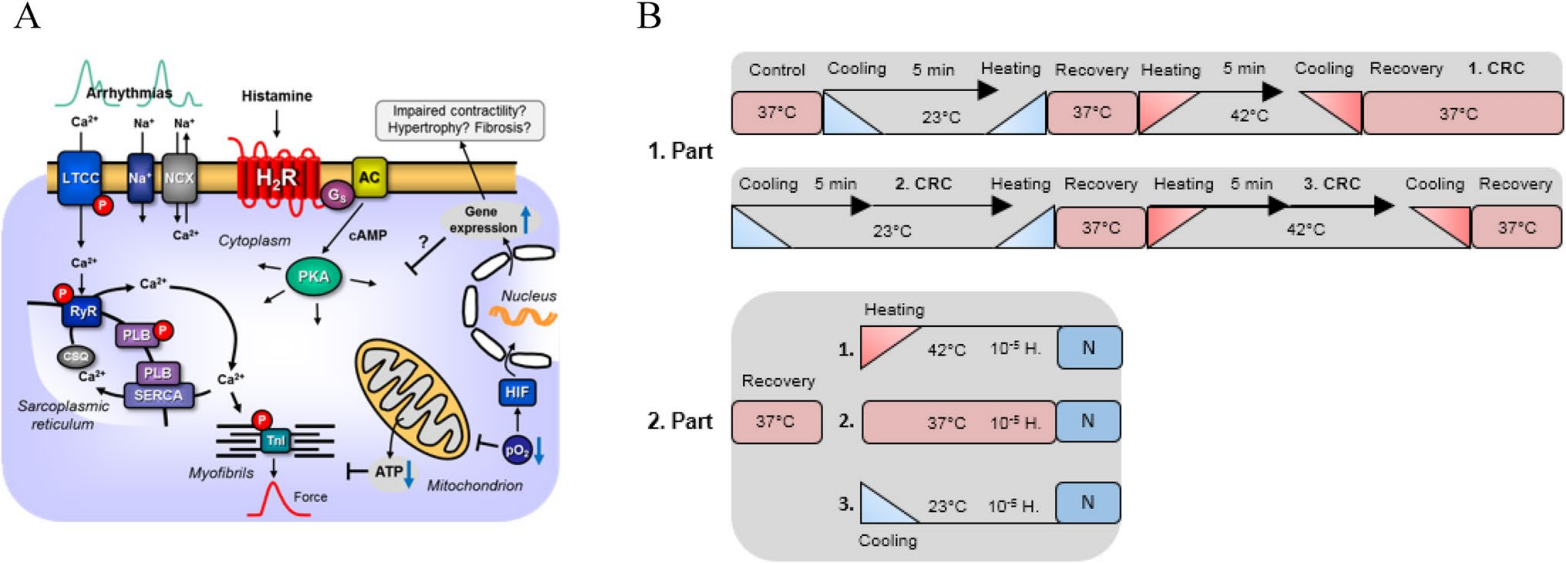
**A** Scheme. Histamine can stimulate H_2_-receptors in cardiomyocytes from H_2_-TG mice. This leads via stimulatory GTP binding proteins (G_s_) to enhanced formation of cAMP by adenylyl cyclase (AC) in the sarcolemma. Produced cAMP then can activate protein kinase (PKA) which usually enhances the activity of regulatory proteins. For instance, enhanced phosphorylation of the inhibitory subunit of troponin (TnI) leads to more rapid relaxation of the heart, whereas enhanced phosphorylation of the L-type Ca^2+^ current (LTCC) leads to increased Ca^2+^ inflow into the cell. This Ca^2+^ can release Ca^2+^ by action on the ryanodine receptor (RyR in the sarcoplasmic reticulum). Release of Ca^2+^ is increased by phosphorylation of RyR. Relaxation is also enhanced by phosphorylation of phospholamban (PLB) which activates SERCA and thereby promotes removal of Ca^2+^. From the vicinity of myofilaments into the sarcoplasmic reticulum. Enhanced entrance of sodium via a sodium channel (Na^+^) can elevate intracellular sodium which can be extruded via the sodium calcium exchanger NCX. Finally, the cell can depolarize and early (EAD) and late afterdepolarization (DAD) could occur manifesting itself as arrhythmias. **B** Protocol of cooling, heating and measuring of concentration response curves (CRC) to histamine. First, for control, without histamine addition at the different temperatures and after that the CRC at the different temperatures. Washing out the histamine was always at 37 °C for recovery. The second part contains the three possibilities after the last CRC

In a previous study, we identified and characterised transgenic mice (labelled H_2_-TG, Fig. 1A) that overexpress the human H_2_ receptor exclusively in the heart via the cardiac-specific α-myosin heavy chain promoter (Gergs et al. 2019a, 2020; Neumann et al. 2021d). The animals (i.e. H_2_-TG mice) responded to histamine administration with elevated atrial and ventricular contractility in vitro and in vivo (Gergs et al. 2019a, 2020; Neumann et al. 2021a), an increased beating rate (Gergs et al. 2019a, 2021a; Neumann et al. 2021c, 2021d) and increased phospholamban (PLB) phosphorylation (mouse: Gergs et al. 2019a) (Fig. 1A). It is clinically significant that histamine increases PLB phosphorylation and augments the phosphorylation state of troponin I in isolated electrically driven human right atrial preparations, indicating that H_2_-TG mice probably employ the same H_2_ receptor signal transduction pathway as humans (Neumann et al. 2021a).

This study addresses the divergent cardiac function of human H_2_ receptors in hypothermia and hyperthermia (Fig. 1A). In both cold and hot environments, humans may experience atrial or ventricular arrhythmias such as ventricular fibrillation (Fig. 1A) (Fukaya et al. 2017; Mackiewicz and Lewartowski 2006; review: Antzelevitch and Yan 2010). Histamine may cause atrial and ventricular cardiac arrhythmias, even under normothermia (review: Neumann et al. 2021b).

Notably, there are similarities between the cardiac effects of serotonin and those of histamine. Like histamine, serotonin can be produced in the heart, especially in the human heart. Serotonin can augment contractile force and quicken the beating rate in human hearts. Serotonin increases the cyclic adenosine monophosphate (cAMP) content and the rate of PLB phosphorylation in the human heart. Like histamine, serotonin does not affect contractility in isolated wild-type atrial preparations. However, we have previously established that there is an affinity for histamine in a transgenic mouse that overexpresses the 5-HT_4_ receptor in the mouse heart. Isolated atrial preparations from this mouse (labelled 5-HT_4_-TG), but not its wild-type (WT) littermate controls, respond to serotonin with increased force generation. We observed that arrhythmias occurred more often in 5-HT_4_-TG right atrial preparations under hypothermia than in their WT littermates (Gergs et al. 2021d). Furthermore, we noticed that the efficacy of serotonin was reduced, while the potency of serotonin was enhanced, in left atrial preparations from 5-HT_4_-TG under hypothermia (Gergs et al. 2021d). Hence, it is an interesting hypothesis that histamine exerts similar inotropic effects as serotonin in a suitable mouse model. Apparently, a comparison with the human atrium may be clinically relevant for translating our findings to the clinical setting.

Therefore, we tested the following hypotheses: (i) hypothermia and hyperthermia alter atrial contractile responses to histamine in humans and in suitable transgenic mice, and (ii) hypothermia and hyperthermia augment atrial arrhythmias in H_2_-TG mice. Previously, progress on this research has been reported solely in the form of abstracts (Neumann et al. 2022; Hoffmann et al. 2022, 2023).

## Materials and methods

### Transgenic mice

We generated the transgenic mice (i.e. H_2_-TG mice) used in this study in a previous study. H_2_-TG mice exhibit cardiac myocyte-specific overexpression of the human H2 receptor via the alpha myosin heavy chain promoter (Gergs et al. 2019a). We handled and maintained the mice with approval and in accordance with the protocols of the Animal Welfare Committee of the Martin Luther University of Halle-Wittenberg, Halle, Germany. We started to generate H_2_-TG because in pilot experiments, histamine under our conditions in the organ bath did not increase force of contraction and the beating rate in isolated atria from wild-type mice (WT). This was the reason why we have generated H_2_-TG: to study histamine and its action on human H_2_-receptors in a mammalian transgenic heart: only in H_2_-TG but not in WT, histamine exerts a positive inotropic effect. We have confirmed this finding repeatedly (Gergs et al. 2019a, b, 2020; Neumann et al. 2021a, b, c). As concerns the biochemical basis for these contractile studies, we detected endogenous mouse H_2_-receptors by polymerase chain reaction (PCR) in hearts from WT (Neumann et al. 2021c). Western blots were done without success (discussed in Gergs et al. a, 2019b): with the commercially available antibodies, we detected only unspecific signals. However, we could detect H_2_-receptors by autoradiography in atria (and ventricles) from H_2_-TG using a radioactive tracer (Gergs et al. 2019a, b) (Table 1).

**Table 1:** Comparison of half-maximal (EC_50_-values) positive inotropic and chronotropic effects of histamine in isolated left atrial preparations (to assess the contraction force) and right atrial preparations (to assess the beating rate) from H_2_-TG. Decadic logarithms of molar drug concentration and SEM are plotted

Temperature	Left atrial force	Frequency	Right atrial force
37 °C	− 7.0 ± 0.1	− 7.1 ± 0.1	− 7.2 ± 0.3
23 °C	− 6.7 ± 0.1*	− 7.2 ± 0.1	− 7.4 ± 0.1
42 °C	− 6.6 ± 0.1*	− 7.1 ± 0.2	− 6.9 ± 0.3

*p < 0.05 vs. 37 °C

### Contractility in isolated atriums from mouse and human

Right and left atrial preparations were prepared and placed in double-barrelled organ baths (Gergs et al. 2013, 2021d; Neumann et al. 2003). We stimulated mouse left atrial preparations and human atrial preparations for 5 ms and 10% over the minimum voltage to initiate the contraction. To start the beating in left atrial preparations of mice, this voltage is usually 5 V. The buffer in the organ bath was initially kept at 37 ℃ (Fig. 1B, 1. Part) using a thermostat (Neumann et al. 1998, 2003; Kirchhefer et al. 2004). After complete stabilisation of the contractile force (which was electrically stimulated at 1 Hz for the left atrial preparations from mice and the human right atrial preparations obtained from humans undergoing cardiac surgery) or the beating rate (in spontaneously beating right atrial preparations from mice), the cardiac temperature was lowered by switching the water supply of the double-barrelled organ baths to a second precooled reservoir. Further details on the differences between changes under hypothermia and hyperthermia have been reported previously and are referred to (Gergs et al. 2021d). The temperature was returned to 37 °C (Fig. 1B), and hyperthermia was induced (Fig. 1B). Subsequently, normothermia was again induced, and a cumulative concentration–response curve to histamine was constructed. Histamine was then washed out, hypothermia was induced and another concentration–response curve to histamine was established. Histamine was washed out after returning the samples to normothermia. Subsequently, hyperthermia was induced and a third concentration–response curve to histamine was constructed. The samples were again returned to normothermia and washed out. Thereafter, the experimental protocol fell into three conditions (Fig. 1B 2. Part): (i) a third of the samples were kept at normothermia, histamine (10 µM) was added, contractile force was recorded and the samples were freeze-clamped with liquid nitrogen for Western blotting; (ii) a third of the atria were subjected to hyperthermia, histamine (10 µM) was added and the atria were freeze-clamped; and (iii) a third of the atria were subjected to hypothermia, histamine (10 µM) was added and the atria were freeze-clamped. The clinical data of patients can be found in Table 2.

**Table 2:** The data of the patients for the human atrial preparations are shown. Including the date, the ejection fraction (EF), diagnosis, the New York Heart Association (NYHA), functional classification and the Canadian Cardiovascular Society (CCS) grading of angina pectoris

Date	EF (%)	Diagnosis	NYHA	CCS
21.04.22	50	3-vessel coronary heart disease	II-III	II-III
27.04.22	35	3-vessel coronary heart disease aortic valve stenosis	III	II-III
02.08.22	50	3-vessel coronary heart disease left main stenosis	III	III
04.08.22	72	3-vessel coronary heart disease left main stenosis	III	III

### Protein expression

Contracting atrial muscle strips from mice and human hearts that were utilised in the atrial organ bath experiments were rapidly brought to the temperature of liquid nitrogen (Gergs et al. 2021d). The samples were kept at – 80 °C and were then subjected to biochemical analysis. Western blotting for serine 16 phosphorylated PLB and the use of calsequestrin as the loading control have been described in a previous study (e.g. Neumann et al. 2021a, 2022). The following primary antibodies were used: anti-serine 16-phosphorylated PLB (anti-PLB-Ser16; 1:5000; Badrilla, Leeds, UK, Cat. # A010-12AP) and anti-calsequestrin (1:20,000; Abcam, Cambridge, UK, Cat. # ab3516). Visualisation of the signal was performed using Immobilon Western chemiluminescent horseradish peroxidase substrate (Merck Millipore, Darmstadt, Germany). We homogenised the cardiac muscle strips, measured the protein concentration in the muscle homogenates, subjected the homogenates to electrophoresis, transferred them to nitrocellulose membranes, incubated the membranes with primary and secondary antibodies and then quantified the results in line with our lab procedures, which have been outlined in several published studies (Gergs et al. 2010, 2019a, b; Boknik et al. 2018, 2019).

### Data analysis

The data are given as the mean ± standard error of the mean. Statistical significance was calculated using an analysis of variance (ANOVA), and we then performed the Bonferroni *t* test or the chi-squared (${\chi }^{2}$) test. Where described in the legend ANOVA, and a Dunnett post hoc test was then performed. We considered a *p* value of < 0.05 as significant. Experimental data on positive agonist-induced inotropic and chronotropic effects were studied by fitting sigmoidal curves to the experimental data using GraphPad Prism 5.0 (GraphPad Software, San Diego, CA, USA). All other statistical analyses were calculated as given in the presented figures and tables, which were also computed using GraphPad Prism 5.0.

### Drugs and materials

Histamine hydrochloride was purchased from Sigma-Aldrich (now Merck), Deisenhofen, Germany. All other chemicals were of the best purity commercially available. 

## Results

We measured the inotropic effect of histamine on human atrial preparations (HAPs) and mouse atrial preparations under normothermia, hypothermia and hyperthermia.

### Human atrial preparations

**Fig. 2:**
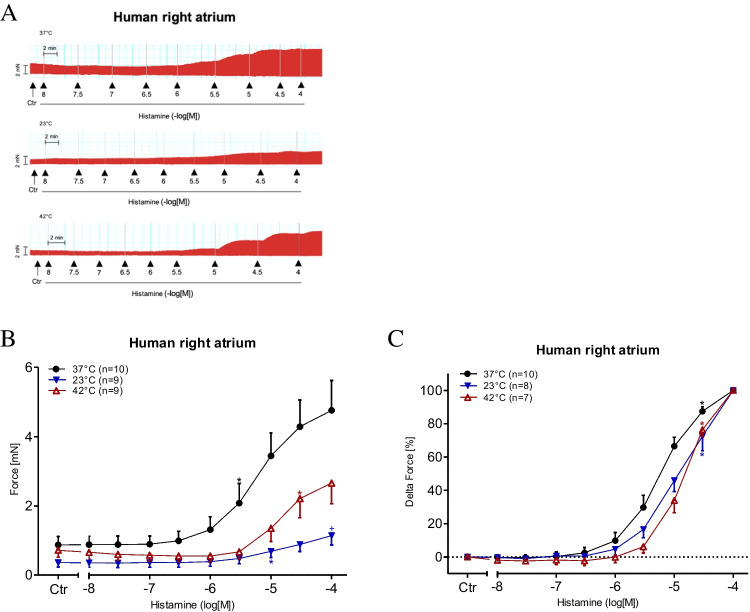
Histamine increases force of contraction in human atrial preparations in a concentration dependent manner. **A** Original recordings of CRC’s in human atrial preparations at the different temperatures (37 °C, 23 °C and 42 °C). Ordinates in **A** and **B** depict developed force of contraction in milli Newton (mN). Ctr (control) indicates force before any drug application. Ordinate in C depicts normalized force: the maximum effect for each concentration response curve is arbitrarily set at 100% and the basal force is set at 0%. Abscissae show negative logarithmic concentrations of histamine. Temperatures of the organ baths are indicated as filled circles (37 °C), filled triangles with tip down (23 °C) or triangles with tip up (42 °C). “*n*” indicates the numbers of experiments. *: first *p* < 0.05 vs. Ctr., + : *p* < 0.05 vs. 37 °C ANOVA Dunnett

In the HAPs, the PIE of histamine was time and concentration dependent; the original data are presented in Fig. 2A. The data on developed tension (measured in mN) are summarised in Fig. 2B. It is apparent that the PIE of histamine on contractile force observed under normothermia is not replicated under either hypothermia or hyperthermia (Fig. 2B). However, the PIE of histamine becomes observable under hypothermia and hyperthermia at higher histamine concentrations than under normothermia (Fig. 2B). Furthermore, the maximum increase in contractile force is lower under hypothermia than under hyperthermia. Hence, it can be argued that low temperatures mitigate the PIE of histamine more strongly than higher temperatures (Fig. 2B). To facilitate a comparison of the different conditions, we arbitrarily set the maximum response to histamine recorded in Fig. 2B as equivalent to 100%. Consequently, it becomes apparent that histamine may be more potent at increasing contractile force at 37 ℃ than at higher or lower temperatures (Fig. 2C). However, a plateau in contractile force was not observed (Fig. 2B); hence, the EC_50_ values could not be properly calculated. We did not use high concentrations of histamine in this study because we failed to wash out histamine at high concentrations in preliminary experiments, and this would have interfered with the intended protocol for measuring all temperature-dependent effects on the same muscle strip (Fig. 1B). Based on our previous experiences, we deemed it essential to compare each muscle strip with itself.

**Fig. 3:**
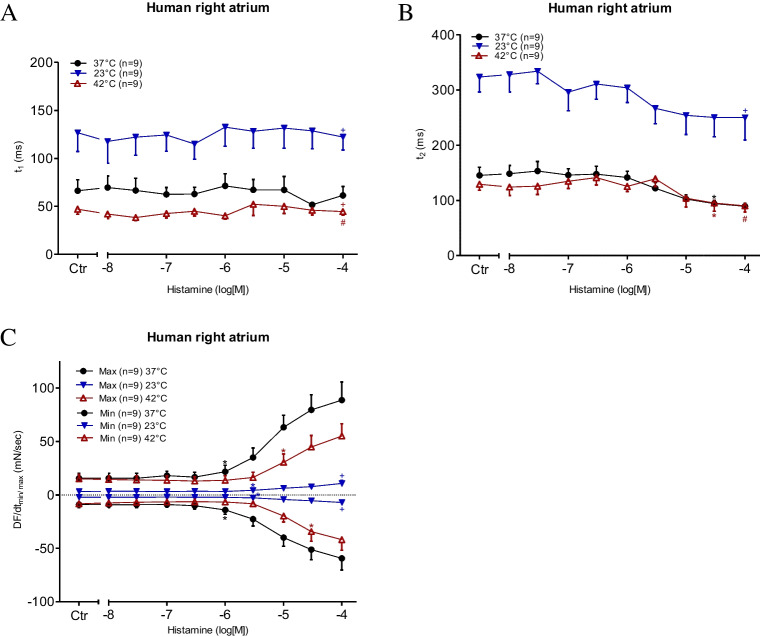
Histamine increases the rate of tension development. Concentration response curves for histamine in human atrial preparation. **C**: dF/dt max and dF/dt min in milli Newton per second (**A**): time to peak tension (t1 in ms) and (**B**): relaxation time (t2 in ms) in electrically driven (1 Hz) right atrial preparations. Abscissae show negative decadic logarithmic concentrations of histamine. Temperatures of the organ baths are indicated as filled circles (37 °C), filled triangles with tip down (23 °C) or triangles with tip up (42 °C). “*n*” indicates the numbers of experiments. *: first *p* < 0.05 vs. Ctr., + : *p* < 0.05 vs. 37 °C ANOVA Dunnett, #: *p* < 0.05 vs. 23 °C ANOVA Dunnett

In the studied samples (Fig. 2), we also assessed the time to peak tension and the time to relaxation (Fig. 3). We observed that the time to peak tension under basal conditions, i.e. the pre-drug values (Ctr), was longer under hypothermia and shorter under hyperthermia than under normothermia (Fig. 3A). However, the basal time to relaxation (Ctr) was the same under normothermia and hyperthermia (Fig. 3B). In contrast, the time to relaxation under basal conditions (Ctr) was much longer under hypothermia than under normothermia (Fig. 3B). Furthermore, histamine concentration dependently decreased the time to relaxation, and this was more marked at low temperatures. However, even the highest histamine concentration did not shorten the absolute values of the time to relaxation recorded under normothermia (Fig. 3B). A particularly clear picture emerged after plotting the maximum positive or negative rate of tension development or relaxation (Fig. 3C). We found that histamine increased the maximum positive or negative rate of tension development or relaxation under normothermia, starting at high control values (no drug administered). In contrast, the changes in the maximum positive or negative rate of tension development or relaxation were smaller under hyperthermia and lowest under hypothermia (Fig. 3C).

### Mouse atrial preparations

**Fig. 4:**
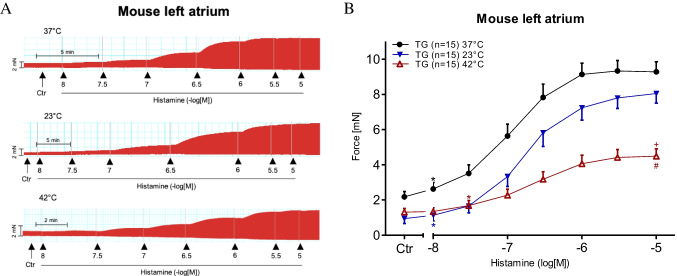
Histamine increases force of contraction in H_2_-TG. **A** Original recordings of effect of increasing concentrations of histamine on electrically stimulated left atrial preparations from H_2_-TG. Concentration dependent effect of histamine on force of contraction. Ordinates in **A** and **B** depict developed force of contraction in milli Newton (mN). Ctr (control) indicates force before any drug application. Abscissae show increasing logarithmic concentrations of histamine. Temperatures of the organ baths are indicated as filled circles (37 °C), filled triangles with tip down (23 °C) or triangles with tip up (42 °C). “*n*” indicates the numbers of experiments. *: first *p* < 0.05 vs. Ctr., + : *p* < 0.05 vs. 37 °C ANOVA Dunnett, #: *p* < 0.05 vs. 23 °C ANOVA Dunnett

**Fig. 5:**
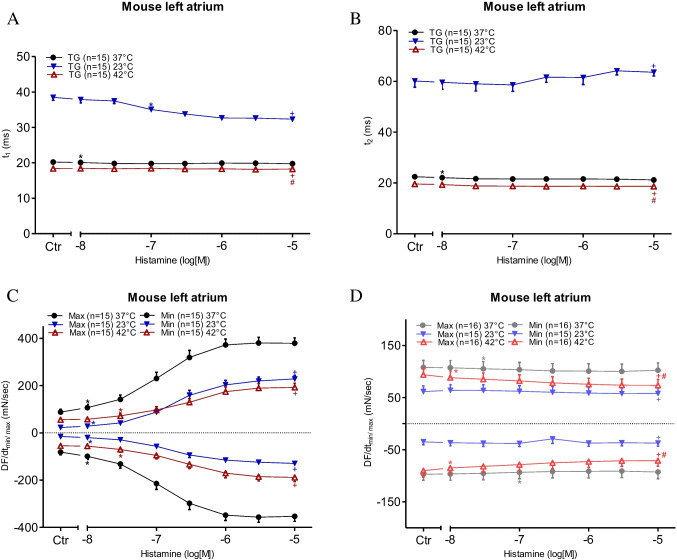
Histamine increases the rate of tension development in left atrium. Concentration response curves for histamine in mouse atrial preparations. **C**, **D** dF/dt max and dF/dt min in milli Newton per second in H_2_-TG (**C**) or WT (**D**), respectively. **A** Time to peak tension (t_1_ in ms) and (B) relaxation time (t_2_ in ms) in electrically driven (1 Hz) left atrial preparations. Abscissae show increasing logarithmic concentrations of histamine. Temperatures of the organ baths are indicated as filled circles (37 °C), filled triangles with tip down (23 °C) or triangles with tip up (42 °C). “*n*” indicates the numbers of experiments. *: first *p* < 0.05 vs. Ctr., + : *p* < 0.05 vs. 37 °C ANOVA Dunnett, #: *p* < 0.05 vs. 23 °C ANOVA Dunnett

To generate a model of the effects of histamine on the human atrium, we studied the effect of histamine on contractile force in left atrial preparations from H_2_-TG mice. The original data on the time-dependent and concentration-dependent PIE of histamine are presented in Fig. 4A. From a comparison of Fig. 4A and Fig. 2A, it is apparent that the inotropic effects of histamine become observable earlier (i.e. at lower concentrations of histamine) in the H_2_-TG mouse left atrial preparations. This is probably why the effect of histamine plateaus in the H_2_-TG mouse left atrial preparations, but not in the HAPs. These two findings are explained by the assumption that the overexpression of the human H_2_ receptor is such that higher values are reached in a mouse atrium (H_2_-TG) than in a human atrium, as discussed in a previous study (Neumann et al. 2021a). Furthermore, in the H_2_-TG mouse left atrial preparations, the maximum positive inotropic effects were higher under normothermia than under hyperthermia and hypothermia (Fig. 4B), the same as in the HAPs (Fig. 2B). For comparison, we also studied the effects of histamine in a WT mice preparation at the three indicated temperatures; however, no PIE of histamine was detected in the WT mice (data not presented). To compare potencies, we calculated EC_50_ values for histamine in H_2_-TG mice at the three temperatures studied. As can be seen in Table 1, histamine was more potent under normothermia than under hypothermia or hyperthermia. Similar to the human atrium (Fig. 3A), the time to peak tension under basal conditions (Ctr) was much longer under hypothermia than under normothermia or hyperthermia (Fig. 5A). Similarly, the time to relaxation under basal conditions (Ctr) was much longer under hypothermia than under normothermia or hyperthermia (Fig. 5B), corresponding with observations in the human samples (Fig. 3B). Histamine in H_2_-TG mice (Fig. 5C) increased both the maximum positive or negative rate of tension development and the rate of relaxation. In contrast, histamine failed to increase the maximum positive or negative rate of tension development or relaxation in the WT mice (Fig. 5D). Furthermore, histamine was less effective at raising the maximum positive or negative rate of tension development or relaxation in H_2_-TG mice under hypothermia and hyperthermia than under normothermia (Fig. 5C), the same as in the HAPs (Fig. 3C). In addition, the maximum positive or negative rates of tension development or relaxation in WT mice were also lower under hypothermia than under normothermia and hyperthermia (Fig. 5D).

**Fig. 6:**
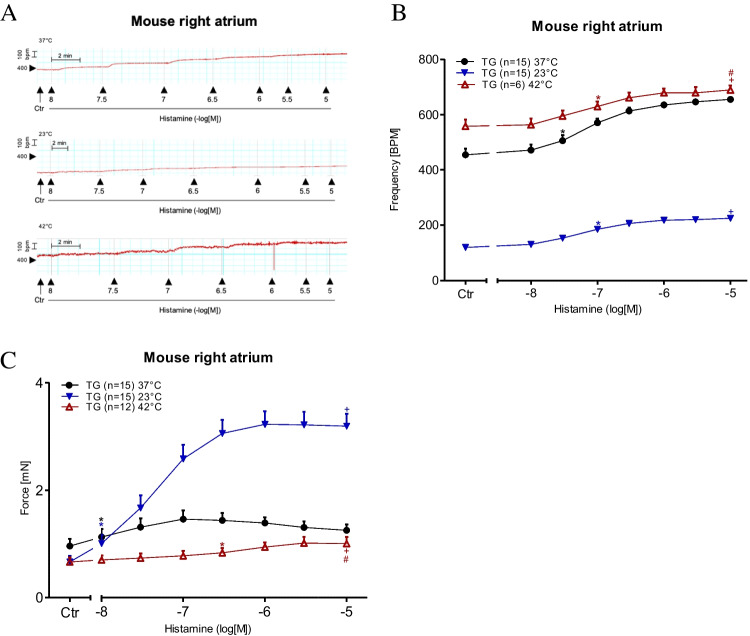
Histamine increases the beating rate and the force of contraction in right atrium. Concentration response curves for histamine in spontaneously beating mouse right atrial preparations. **A** Original recordings of effect of increasing concentrations of histamine in the beating rate, **B** beating rate in beats per minute (BPM), **C** force of contraction in milli Newton. Abscissae show increasing logarithmic concentrations of histamine. Temperatures of the organ baths are indicated as filled circles (37 °C), filled triangles with tip down (23 °C) or triangles with tip up (42 °C). “*n*” indicates the numbers of experiments. *: first *p* < 0.05 vs. Ctr., + : *p* < 0.05 vs. 37 °C ANOVA Dunnett, #: *p* < 0.05 vs. 23 °C ANOVA Dunnett

**Fig. 7:**
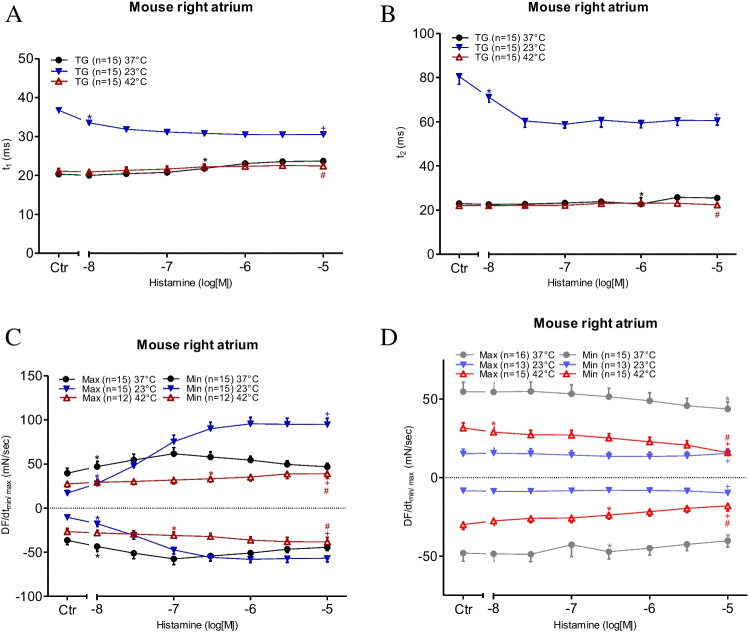
Hyperthermia and hypothermia reduce the effect of histamine on tension development in right atrial preparations. **C**, **D** dF/dt max and dF/dt min in milli Newton per second in H_2_-TG (**C**) or WT (**D**), respectively. **A** Time to peak tension (t_1_ in ms) and **B** relaxation time (t_2_ in ms) in spontaneously beating right atrial preparations. Abscissae show increasing logarithmic concentrations of histamine. Temperatures of the organ baths are indicated as filled circles (37 °C), filled triangles with tip down (23 °C) or triangles with tip up (42 °C). “*n*” indicates the numbers of experiments. *: first *p* < 0.05 vs. Ctr., + : *p* < 0.05 vs. 37 °C ANOVA Dunnett, #: *p* < 0.05 vs. 23 °C ANOVA Dunnett

For comparison against the findings from the left mouse atrium, we also studied the spontaneous beating rate in isolated right atrial preparations. Remarkably, under basal conditions (Ctr = no drug administered), the beating rate was higher under hyperthermia than under normothermia and lower under hypothermia. The original data are presented in Fig. 6A, which shows that histamine potently increased the beating rate (Fig. 6B). However, the EC_50_ values for histamine PCE in H_2_-TG mice were not altered by changes in temperature (Table 1). We further assessed the contractile force developed in the right atrium (Fig. 6C). As described earlier, histamine augmented the contractile force in the left atrium in a more concentration-dependent manner than in the right atrium (Fig. 6C). The EC_50_ values are summarised in Table 1. The maximum PIE of histamine was much lower under normothermia than under hypothermia, and there was practically no PIE of histamine under hyperthermia (Fig. 6C). Similar to the left atrial preparations, the time to peak tension under basal conditions (Ctr) in the right atrial preparations was more significantly enhanced by hypothermia than by normothermia or hyperthermia (Fig. 7A). Histamine under hypothermia brought about a concentration-dependent shortening of the time to peak tension (Fig. 7A) and the time to relaxation (Fig. 7B). Similar to the left atria (Fig. 5B), the time to relaxation in the right atria under basal conditions (Ctr) was much longer at lower temperatures than at normal or higher temperatures (Fig. 7B). Furthermore, much like in the left atrial preparations (Fig. 5C), histamine increased the maximum positive or negative rate of tension development or relaxation in the right atrial preparations from H_2_-TG mice (Fig. 7C), but no such effect was noticeable in WT mice (Fig. 7D) (Table 3).

**Table 3:** Basal parameters of the human and mouse atrial preparations (*n* = 6–15)

Human right atrium	37 °C	23 °C	42 °C
basal tension (mN)	0.87 ± 0.25	0.36 ± 0.13	0.72 ± 0.20
basal time-to-peak tension (ms)	66.2 ± 11.5	127 ± 19.8*	47.1 ± 5.36
basal relaxation time (ms)	145 ± 14.5	311 ± 26.9*	125 ± 10.5
Mouse left atrium			
basal tension (mN)	2.18 ± 0.30	0.94 ± 0.28*	1.31 ± 0.21*
basal time-to-peak tension (ms)	20.4 ± 0.22	38.4 ± 0.82*	18.5 ± 0.16*
basal relaxation time (ms)	23.1 ± 0.78	63.0 ± 3.04*	19.7 ± 0.80*
Mouse right atrium			
basal tension (mN)	0.96 ± 0.13	0.67 ± 0.11*	0.67 ± 0.08*
basal time-to-peak tension (ms)	20.4 ± 0.40	36.8 ± 0.64*	21.1 ± 0.72
basal relaxation time (ms)	23.1 ± 0.54	80.6 ± 3.57*	22.0 ± 0.80
basal beating rate (bpm)	454 ± 22.2	120 ± 10.4*	534 ± 3 2.1*

^*^p < 0.05 vs. 37 °C

### Protein phosphorylation

**Fig. 8:**
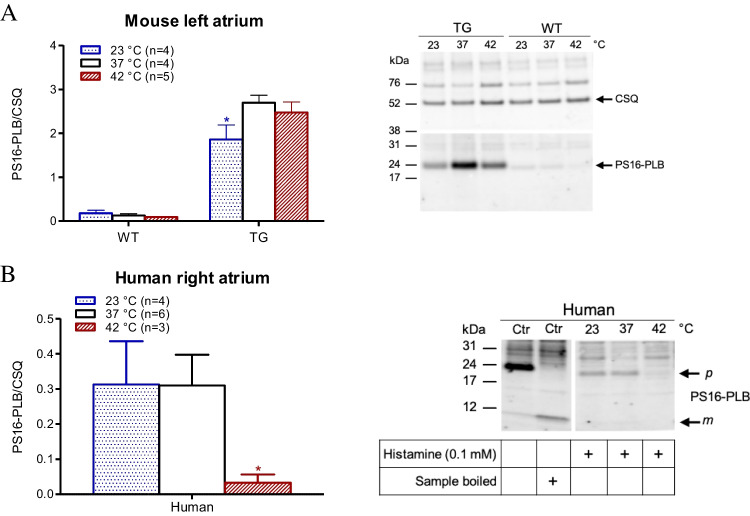
The effect of histamine on phosphorylation of phospholamban is temperature dependent. **A**. **B** Right-hand side: original Western blots: height of samples is given in kDA by arrows for protein markers. Western blots depicting expression serine 16 phosphorylated phospholamban and calsequestrin (CSQ) as loading control in wild type (WT) and H_2_-TG (TG) hearts are shown. The human control samples (Ctr) were treated with isoprenaline. Expression data were quantified and normalized to CSQ expression (ordinates). Temperatures of the organ baths are indicated as white bars (37 °C), dotted bars (23 °C) and dashed bars (42 °C). “*n*” indicates the numbers of experiments. *p < 0.05 vs. 37 °C

Next, we were curious about how PLB phosphorylation in the H_2_-TG mice and human preparations was altered under our experimental conditions. As can be seen in Fig. 8A, histamine (10 µM) increased the phosphorylation state of PLB at amino acid serine 16 in H_2_-TG mice (Fig. 8A), in the same way it increased the rate of relaxation (Fig. 5C). Apparently, the relaxant effect of histamine is less potent under hypothermia than under hyperthermia or normothermia (Fig. 5C), and this difference is mirrored by the increase in PLB phosphorylation being less under hypothermia than under normothermia, which is consistent with a causal relationship between the extent of PLB phosphorylation and the time to relaxation. However, histamine did not increase the phosphorylation state of PLB at amino acid serine 16 in WT mice (Fig. 8A), which corresponds with histamine having no inotropic or lusitropic effect in WT mice (Fig. 5). Interestingly, under hypothermia, histamine effected a smaller increase in the phosphorylation state of PLB at amino acid serine 16 than it did under normothermia (Fig. 8A). The controls for identifying PLB are presented in Fig. 8B: the first two lanes represent positive controls, with the effect of a maximally effective concentration of isoprenaline (1 µM) on protein phosphorylation in the first lane; and in the neighbouring lane is the similar sample but boiled before application to the gel. Here, PLB exhibits a typical mobility shift from a pentameric to monomeric form, facilitating the identification of these bands as phosphorylated PLB. Lastly, we studied the phosphorylation state of PLB at amino acid serine 16 in HAPs (Fig. 8B). The histamine-induced increase in the phosphorylation state of PLB at amino acid serine 16 in HAPs (Fig. 8B) was less under hyperthermia than under normothermia.

### Arrhythmias

**Fig. 9:**
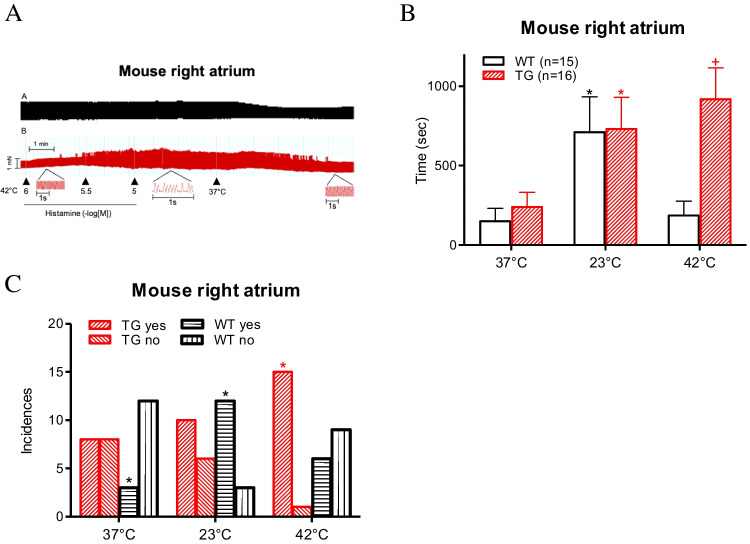
Hyperthermia leads to arrhythmias. Occurrence of arrhythmias during at the indicated temperatures in right atrial preparations from WT and H_2_-TG mice. **A** Original recordings of right atrial preparations from WT (**A**) and H_2_-TG (**B**) mice during hyperthermia protocol. **B** Temperature-dependent average duration of arrhythmias during histamine addition in WT (white bar) and H_2_-TG (dashed bar). Ordinate depicts the time in seconds and the abscissa shows the different temperatures. “*n*” indicates the numbers of experiments. *p < 0.05 vs. 37 °C. + *p* < 0.05 vs. WT. **C** Temperature-dependent incidences of arrhythmias during histamine addition in WT (horizontal and vertical dashed bars) and H_2_-TG (diagonal dashed bars). Ordinate shows the incidences of the arrhythmias. The number of experiments were *n* = 16. *p < 0.05 (${\chi }^{2}$-test)

Next, we addressed the question of whether the incidence and duration of arrhythmias are affected by temperature in H_2_-TG mice. We present typical original recordings of muscle preparations from the right atrium in Fig. 9A. The insets show the contractile force at a high temporal resolution to facilitate the recognition of arrhythmias. Arrhythmias typically developed shortly after histamine addition under hyperthermia (Fig. 9A). Under basal conditions, arrhythmias were observed to occur—when there had previously been no arrhythmias—when they were histamine-induced. These arrhythmias were reversible after cooling the organ bath back to 37 °C (Fig. 9A). The results of these experiments are plotted in bar diagrams, from which it is apparent that, under hyperthermia, the duration of arrhythmias was longer in H_2_-TG mice than in WT mice (Fig. 9B). Furthermore, arrhythmias occurred more often under hyperthermia in H_2_-TG mice than in the WT mice (Fig. 9C).

## Discussion

An especially critical finding in this study is that hyperthermia leads to a higher incidence and duration of cardiac arrhythmias in mouse right atrial preparations with overexpressed human H_2_ receptors than in WT mice. These arrhythmias were reversible when the temperature was lowered and returned to normothermia (37 °C), suggesting that the effect is due to hyperthermia, specifically. Furthermore, we found that the potency and efficacy of histamine at augmenting contractility and heart rate in H_2_-TG mice were diminished under hyperthermia. Based on these data, we propose that hyperthermia alters the signal transduction path of the H_2_ receptor.

In addition, under hyperthermia, arrhythmias were observed more often in H_2_-TG mice than in WT mice. Presently, we can only speculate about the mechanisms involved. Interestingly, in left atrial preparations, histamine-stimulated PLB phosphorylation was less at temperatures lower than 37 °C. In contrast, we explain in a previous study that there is an increased basal incidence of arrhythmias at 37 °C in H_2_-TG mice under basal conditions and normothermia, which is induced by an elevated basal generation of cAMP that subsequently increases the phosphorylation state of PLB (Fig. 1A) (Gergs et al. 2021c). Other targets that could potentially produce arrhythmias under hyperthermia include altered potassium currents, altered L-type Ca^2+^ currents, inhibited sodium channel functions and altered Ca^2+^ content in the sarcoplasmic reticulum (Gregory and Weant 2021; El-Battrawy et al. 2016; Zhao et al. 2016; Abdelsayed et al. 2015; Morita et al. 2007; Keller et al. 2006).

One can compare our findings using histamine with studies using isoproterenol. Isoproterenol directly stimulates β-adrenoceptors. Thence, isoproterenol should use the cAMP system to increase force and beating rate in the mouse or human heart like histamine in this context (Fig. 1). We noted in left atrial preparations from WT that isoproterenol tended to be less potent to raise force of contraction at 23 °C than at 37 °C (Gergs et al. 2021a, b, c, d). Similarly, the effect of histamine on force of contraction in H_2_-TG at 23 °C is located to the right of the curve at 37 °C (Fig. 4B). Hence, we could tentatively conclude that a similar mechanism might explain the effects of histamine and isoproterenol at lower temperature. A similar pattern was noted by others before: the curve for the positive inotropic effect of isoproterenol was shifted to the right at 25 °C compared to 37 °C in the isolated electrically stimulated guinea pig left atrial preparation (Tenner and McNeill 1978; Reinhardt et al. 1978). The situation in the right atrial preparations was different: in the atrial preparations form WT, hypothermia reduced the spontaneous beating rate (Gergs et al. 2021a, b, c, d) but the positive chronotropic effect of isoproterenol was of similar potency at 23 °C and at 37 °C (Gergs et al. 2021a, b, c, d). In a similar pattern, the spontaneous beating rate was lower at 23 °C compared to 37 °C but increased similarly at both temperatures in H_2_-TG after addition of histamine (Fig. 6B).

As concerns hyperthermia, at least in isolated left atrial preparations from the guinea pig the inotropic effects of isoproterenol were greatly attenuated (efficacy and potency reduced: Reinhardt et al. 1978). These findings are in agreement with our findings in human atrial muscle strips (Fig. 2B) and in left atrial preparations from H_2_-TG (Fig. 4B) and right atrial preparations from H_2_-TG (Fig. 6C).

Heat (42 °C) reduced contractile force generation in isolated left atrial preparations and elevated the spontaneous rate of contraction in right atrial preparations from H_2_-TG mice. Furthermore, it is noteworthy that the PIE of histamine was attenuated in H_2_-TG mice at 42 °C. This may be in agreement with the research findings of others, such as the inotropic effect to histamine in guinea pig papillary muscles (via H_2_ receptors) being lower at 25 °C than at 37.5 °C (papillary muscle from guinea pig and rabbit: Longhurst and McNeill 1983). Similarly, in rabbit mesenteric arteries, histamine was less potent at exerting a relaxant effect via H_2_ receptors at 25 °C than at 42 °C (via H_2_ receptors in mesenteric arteries: Reinhardt and Ritter 1979).

In human atrial preparations, a complete concentration response curve using up to 100 µM histamine as the highest concentration led under our standard experimental conditions (37 °C) to a plateau and we then obtained for the positive inotropic effect a pEC_50_-value of 5.11 ± 0.32 (*n* = 9, recalculated from Neumann et al. 2021a, b, c, d). Others reported similar pEC_50_-values for histamine in human atrial preparation of 5.54 (Zerkowski et al. 1993). Hence, these EC_50_-values show that the concentration–response curve in the present paper at 37 °C (Fig. 2B) is in reasonable agreement with the literature.

### Protein phosphorylation

We mentioned earlier that histamine augments PLB phosphorylation under normothermia in isolated cardiac preparations obtained from H_2_-TG mice, as published in a previous study (Gergs et al. 2019a, b). Under hypothermia, the same concentration of histamine increased the phosphorylation state of PLB less than it did under normothermia. Presently, we surmise that cAMP production is less at temperatures lower than those at normothermia because the H_2_ receptor in H_2_-TG mice is less active at low temperatures; notably, this is the inverse of our findings with human samples. Histamine increases PLB phosphorylation at 37 ℃, but barely affects PLB phosphorylation under hyperthermia. Moreover, one has to keep in mind that the monomer of PLB is more effective to inhibit the activity of SERCA (Fig. 1) than the pentameric form (review: Kadambi and Kranias 1997). Thus, one might speculate that Hence, a possible explanation would be that the H_2_ receptor in the human heart is less capable of coupling under hyperthermia than under normothermia. Moreover, β-adrenergic stimulation of the heart also leads to phosphorylation of PLB on threonine 17 ((review: Kadambi and Kranias 1997). We had published that threonine 17 phosphorylation of PLB is stimulated by histamine in hearts from H_2_-TG (Gergs et al. 2019a, b). Hence, a second pathway of histamine action on PLB has to be considered. However, whether under our conditions the basal phosphorylation of PLB at threonine 17 is also temperature dependent (Fig. 8A) needs to be elucidated. We overexpressed the human H_2_ receptor in the mice; hence, it would be puzzling if the sole explanation for these differences (Fig. 8) were to lie in the receptor itself. However, coupling to other proteins may be species-dependent, which might explain our observation. Theoretically, cAMP-dependent protein kinases (Kovalevsky et al. 2012) and protein phosphatases (PP2A: Yorimitsu et al. 2009) may exhibit species-dependent sensitivity to temperature, but this is a topic for future research.

## Study limitations

We did not observe arrhythmias in the HAPs. This might be because the HAPs were electrically stimulated, as they did not contain the human sinus node. Fittingly, we did not detect any arrhythmias in the electrically driven mouse (left) atrial preparations. Perhaps, we need spontaneously beating human atrial cells for this series of experiments. Although these cells can now be generated from pluripotent human stem cells, this option was beyond the scope of this study. Moreover, our data are mainly descriptive. In future work, it should be the aim to present a direct molecular mechanism why histamine leads to arrhythmias via H_2_-receptors in hyperthermia. Furthermore, in a previous study, we noted more arrhythmias at hypothermia than at normothermia in isolated right atrial preparations from mice that overexpress the human 5-HT_4_-receptor (Gergs et al. 2021a, b, c, d). Theoretically, both receptors (H_2_-receptor and 5-HT_4_-receptor) signal through the cAMP system and thus similar phenotypes (arrhythmias) might be expected. However, one hypothetical explanation might be that subtle signal transduction differences might exist. For instance, stimulation of non-canonical pathways (via arrestins) might differ between the receptors. But more work in this respect is clearly required.

In summary, in right atrial preparations from H_2_-TG mice, hyperthermia increases the beating rate and may induce arrhythmias. The incidence of hyperthermia-induced arrhythmias was more frequent in H_2_-TG mice than in the WT mice. Thus, we propose that H_2_ receptors may be at the root of atrial fibrillations in patients experiencing hyperthermia (e.g., in the form of fever) and that H_2_ receptor antagonists can be used to terminate such arrhythmias.

## Data Availability

The data of this study are available from the corresponding author upon reasonable request.
